# Remdesivir targets a structurally analogous region of the Ebola virus and SARS-CoV-2 polymerases

**DOI:** 10.1073/pnas.2012294117

**Published:** 2020-10-07

**Authors:** Michael K. Lo, César G. Albariño, Jason K. Perry, Silvia Chang, Egor P. Tchesnokov, Lisa Guerrero, Ayan Chakrabarti, Punya Shrivastava-Ranjan, Payel Chatterjee, Laura K. McMullan, Ross Martin, Robert Jordan, Matthias Götte, Joel M. Montgomery, Stuart T. Nichol, Mike Flint, Danielle Porter, Christina F. Spiropoulou

**Affiliations:** ^a^Viral Special Pathogens Branch, US Centers for Disease Control and Prevention, Atlanta, GA 30329;; ^b^Gilead Sciences Inc., Foster City, CA 94404;; ^c^Department of Medical Microbiology and Immunology, University of Alberta, Edmonton, AB T6G 2E1, Canada;; ^d^Li Ka Shing Institute of Virology, University of Alberta, Edmonton, AB T6G 2E1, Canada

**Keywords:** remdesivir, Ebola, COVID-19, SARS-CoV-2, antiviral nucleotide analog

## Abstract

Remdesivir is a broad-spectrum antiviral nucleotide prodrug that has been clinically evaluated in Ebola virus patients and recently received emergency use authorization (EUA) for treatment of COVID-19. With approvals from the Federal Select Agent Program and the Centers for Disease Control and Prevention’s Institutional Biosecurity Board, we characterized the resistance profile of remdesivir by serially passaging Ebola virus under remdesivir selection; we generated lineages with low-level reduced susceptibility to remdesivir after 35 passages. We found that a single amino acid substitution, F548S, in the Ebola virus polymerase conferred low-level reduced susceptibility to remdesivir. The F548 residue is highly conserved in filoviruses but should be subject to specific surveillance among novel filoviruses, in newly emerging variants in ongoing outbreaks, and also in Ebola virus patients undergoing remdesivir therapy. Homology modeling suggests that the Ebola virus polymerase F548 residue lies in the F-motif of the polymerase active site, a region that was previously identified as susceptible to resistance mutations in coronaviruses. Our data suggest that molecular surveillance of this region of the polymerase in remdesivir-treated COVID-19 patients is also warranted.

## Significance

Remdesivir is a nucleotide analog prodrug that has been evaluated in humans against acute Ebola virus disease; it also recently received emergency use authorization for treating COVID-19. For antiviral product development, the Food and Drug Administration recommends the characterization of in vitro selected resistant viruses to define the specific antiviral mechanism of action. This study identified a single amino acid residue in the Ebola virus polymerase that conferred low-level resistance to remdesivir. The significance of our study lies not only in characterizing this particular mutation, but also in relating it to a resistance mutation observed in a similar structural motif of coronaviruses. Our findings thereby indicate a consistent mechanism of action by remdesivir across genetically divergent RNA viruses causing diseases of high consequence in humans.

Remdesivir (GS-5734) is a nucleotide analog prodrug that has been clinically evaluated against both Ebola virus disease (EVD) and COVID-19, and has recently received emergency use authorization (EUA) for the latter (1, 2). Remdesivir was first characterized and evaluated as a potent inhibitor of Ebola virus (EBOV) (*Zaire ebolaviruses* species) amid the historically largest EVD epidemic (3–6), and was then also evaluated during the second largest EVD epidemic in Democratic Republic of Congo (DRC) (1, 7). Remdesivir has also shown efficacy in mice and nonhuman primates against other highly pathogenic respiratory pathogens, including Nipah virus (NiV) and both severe acute respiratory syndrome coronavirus (SARS-CoV-1) and Middle East respiratory syndrome coronavirus (MERS-CoV) (8–10). Furthermore, preliminary evidence from clinical evaluations indicate that remdesivir shortens the recovery time of hospitalized COVID-19 patients presumably by blocking RNA replication of severe acute respiratory syndrome coronavirus 2 (SARS-CoV-2) (11). Remdesivir has been biochemically shown to inhibit the activity of EBOV large (L) RNA-dependent RNA polymerase (RdRp) as a nonobligate delayed chain terminator (12). We sought to further understand the mechanism of EBOV inhibition and to identify determinants of resistance that may arise upon treatment of patients with remdesivir. Importantly, such determinants may naturally be present in other filoviruses, either known or yet to cross over into the human population. We serially passaged recombinant EBOVs with subclinical concentrations of remdesivir and demonstrated the reduced susceptibility of these viruses to remdesivir after 35 passages. We identified a single-nucleotide variant (SNV) that emerged across six independent remdesivir-selected EBOV lineages; this mutation resulted in a nonconservative amino acid substitution at residue 548 (F548S) in the fingers subdomain of the EBOV L RdRp. We examined this mutation in several contexts: a cell-based minigenome, a cell-free biochemical polymerase assay, as well as in a full-length infectious recombinant EBOV. In the context of the infectious virus, the F548S substitution recapitulated the reduced susceptibility phenotype to remdesivir, and potentially showed a marginal decrease in viral fitness compared to wild type. Thus, our study importantly identifies a molecular marker for reduced remdesivir susceptibility.

Guidance from the Food and Drug Administration on antiviral product development recommends the characterization of in vitro-selected resistant viruses to define the mechanism of action and establish the specific antiviral activity. Although this is done routinely during the preclinical development of new antiviral therapies, to our knowledge, this has not been done for a candidate anti-EBOV agent. Our findings have implications for the surveillance of filovirus sequences, for treatment of EBOV patients with remdesivir or other similarly acting inhibitors, and for the development of future anti-EBOV therapies. Furthermore, comparative structural modeling of the EBOV and SARS-CoV-2 RdRp domains indicate remdesivir targets the polymerases of EBOVs and CoVs similarly, such that our findings may have implications for remdesivir treatment of COVID-19 patients.

## Results

### Serial Passaging of Recombinant Reporter Ebola Virus under Remdesivir Selection Resulted in Fourfold to Fivefold Reduced Susceptibility.

**Fig. 1.**
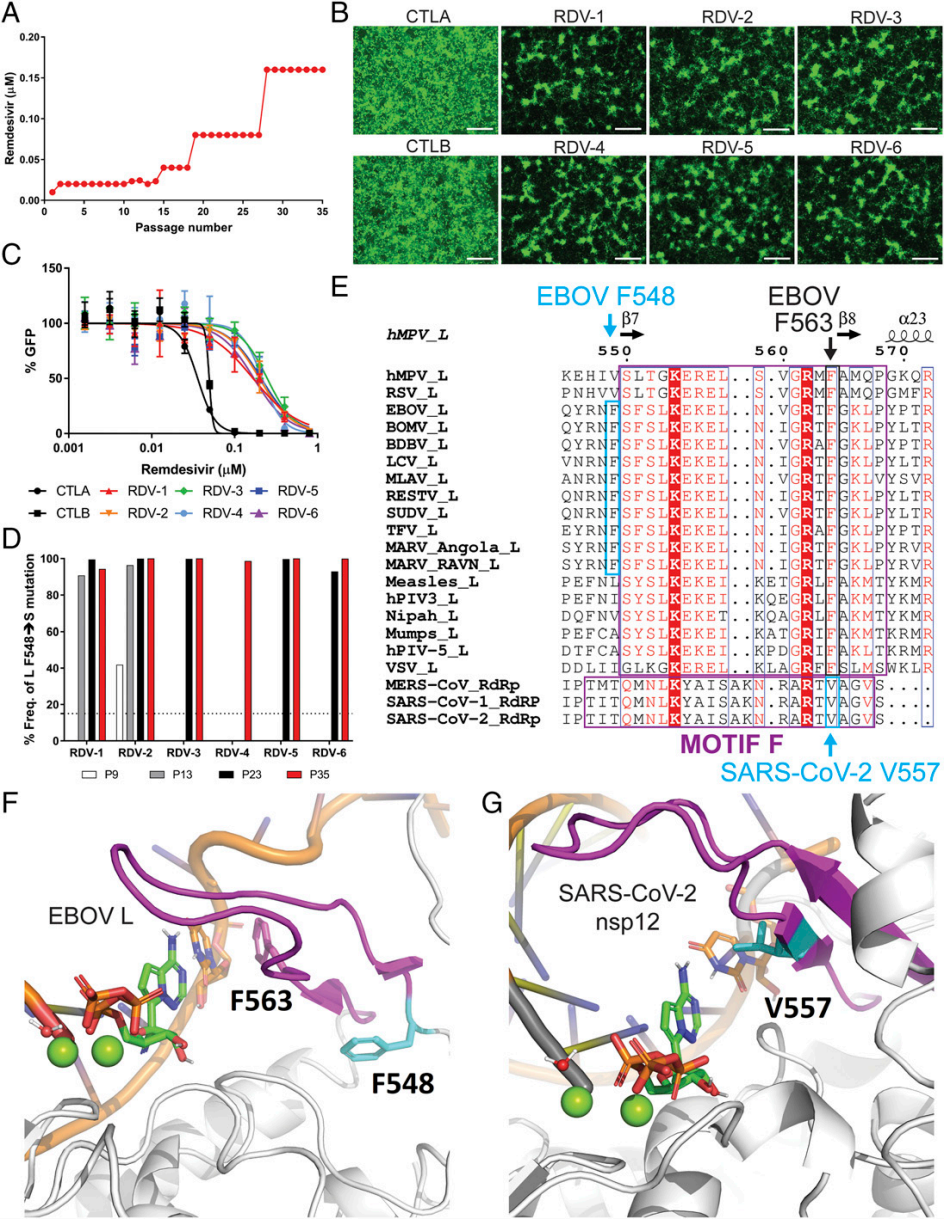
Serial passaging under remdesivir selection confers reduced susceptibility of rEBOV/ZsG to remdesivir correlated with a single amino acid change in the RNA-dependent RNA polymerase (RdRp) domain of the EBOV L polymerase. (*A*) Graphical plot of remdesivir concentrations used over 35 serial passages (range, 0.01 to 0.16 µM). (*B*) Fluorescence micrographs of passage 35 mock-selected control EBOV/ZsG (CTLA/B) or remdesivir-selected rEBOV/ZsG (RDV-1 through RDV-6) in Huh7 cells taken 96 h postinfection (hpi) at 4× magnification; white bar indicates 500 μm. (*C*) Remdesivir inhibition of green fluorescence signal (percentage GFP) generated by passage 35 mock-selected (black symbols) and remdesivir-selected (colored symbols) rEBOV/ZsG lineages at 144 hpi. Error bars indicate SD from the mean of duplicate samples. (*D*) Frequencies of single-nucleotide variant (SNV) resulting in EBOV L F548S amino acid coding sequence change detected among remdesivir-selected rEBOV/ZsG viruses at passages 9 (white bar), 13 (gray bars), 23 (black bars), and 35 (red bars) are plotted. The dotted line indicates 15% SNV frequency cutoff. (*E*) Structure-based protein alignment of L polymerases from viruses across the order *Mononegavirales*, and also the RdRps of betacoronaviruses MERS-CoV, SARS-CoV-1, and SARS-CoV-2 (GenBank accession numbers used for this alignment are found in *Materials and Methods*). The secondary structures of the human metapneumovirus L polymerase hMPV-L are displayed above the alignment. Evolutionarily conserved F motifs are respectively boxed in violet, while the F548 residue conserved across respective filovirus polymerases are boxed in cyan. (*F*) The putative structure of the EBOV L polymerase in its replicating state was modeled based on the defined structures of the human metapneumovirus (PDB ID code 6U5O) (14) and respiratory syncytial virus (PDB ID code 6PZK) (15) polymerases. The side chain of amino acid residue F548 is shown in cyan; the side chain of residue F563 (analogous to SARS-CoV-2 V557) is shown in violet as part of conserved motif F. (*G*) The model of preincorporated RDV-TP in SARS-CoV-2 nsp12 was based on the defined structure of the SARS-CoV-2 replication complex nsp12–nsp7–nsp8 with dsRNA (PDB ID code 6YYT) (19). The side chain of amino acid residue V557 is shown in cyan.

Approval to conduct this study was received from the Centers for Disease Control and Prevention (CDC) Institutional Biosecurity Board and the Federal Select Agent Program. We conducted serial passaging experiments using recombinant EBOV that expresses a ZsGreen1 (ZsG) fluorescent reporter protein (rEBOV/ZsG) (13) to facilitate monitoring of viral replication. We concurrently passaged two independent mock-selected rEBOV/ZsG lineages (CTLA and CTLB) alongside six independent remdesivir-selected rEBOV/ZsG lineages (RDV-1 through RDV-6). The initial remdesivir selection concentration used was the previously defined 50% effective inhibition concentration (EC_50_) of ∼0.01 µM (3–5). Following the initial passage, remdesivir concentrations were raised to ∼0.02 to 0.025 µM for 14 consecutive passages, then to 0.04 µM for 4 passages, 0.08 µM for an additional 9 passages, and finally to a maximum of 0.16 µM for the final 8 passages (Fig. 1*A*). Fluorescence micrographs were taken for each lineage at each passage, and the infectious yields from virus supernatants were measured by 50% tissue culture infectious dose (TCID_50_) assay at each passage (*SI Appendix*, Fig. S1). By the 35th serial passage, rEBOV/ZsG replication and spread was apparent across all six remdesivir-selected lineages, albeit at visibly lower levels than observed for the two mock-selected lineages (Fig. 1*B*). We then conducted dose–response inhibition assays against remdesivir for both remdesivir-selected and mock-selected lineages and measured their respective EC_50_ values based on reporter fluorescence levels (Fig. 1*C* and Table 1). Compared to the mock-selected lineages, all six of the remdesivir-selected lineages had notably higher EC_50_ values, showing 3.7- to 5.2-fold reduced susceptibilities to remdesivir inhibition (Table 1).

**Table 1.** Susceptibility of mock-selected and remdesivir-selected rEBOV/ZsG lineages to remdesivir after 35 serial passages

Virus ID	Remdesivir EC_50_, µM	Fold change over CTLA/B
CTLA/B	0.046 ± 0.011	1
RDV-1	0.22 ± 0.049	4.8
RDV-2	0.23 ± 0.028	5
RDV-3	0.23 ± 0.016	5
RDV-4	0.17 ± 0.049	3.7
RDV-5	0.24 ± 0.026	5.2
RDV-6	0.22 ± 0.054	4.8

Mean EC_50_ values were calculated from reporter assays conducted 144 hpi. Means and SDs are derived from two independent experiments performed in duplicate and quadruplicate.

### A Single-Nucleotide Variant Detected in All Remdesivir-Selected Passage 35 rEBOV/ZsG Lineages Resulted in an Amino Acid Substitution within the RdRp Domain of the L Polymerase Gene.

We conducted next-generation sequencing (NGS) of all passage 35 mock-selected and remdesivir-selected rEBOV/ZsG lineages to identify SNVs that correlated with the reduced susceptibility of the remdesivir-selected lineages. Using a 15% SNV frequency cutoff, we detected four SNVs resulting in coding sequence changes that were found in at least two of the six remdesivir-selected lineages but were not present in mock-selected lineages, at frequencies ranging from 39 to 100% (Table 2). Three of the four SNVs were in the L polymerase gene coding sequence, whereas the remaining SNV resided in the multifunctional polymerase cofactor (VP35) gene. Of the three L gene SNVs, the SNV at nucleotide position 13984 of the rEBOV/ZsG genome was detected across all six remdesivir-selected lineages with a minimum frequency of ∼94%. This SNV resulted in a nonconservative phenylalanine to serine substitution at amino acid position 548 (F548S).

**Table 2.** Single-nucleotide variants resulting in coding sequence changes detected in two or more remdesivir-selected passage 35 rEBOV/ZsG lineages

	Gene and SNV ID	VP35 G4056A	L T13984C	L A14025G	L A14620G
Virus ID	CDS change	S310N	F548S	T562A	E760V
RDV-1	% SNV freq.		94.31	62.61	
	read depth		562	591	
RDV-2	% SNV freq.	97.99	100	52.57	
	read depth	1,197	583	1,090	
RDV-3	% SNV freq.		100	73.51	
	read depth		666	823	
RDV-4	% SNV freq.	39.23	98.67		
	read depth	181	75		
RDV-5	% SNV freq.		100	54.53	52.76
	read depth		236	237	326
RDV-6	% SNV freq.		99.89		47.37
	read depth		934		1,503

Reference sequence for rEBOV/ZsG: GenBank accession number KR781609.1. Freq., frequency. Cutoff frequency for SNV detection, 15%. CDS, coding sequence; SNV, single-nucleotide variant.

To identify when the F548S mutation emerged, we sequenced remdesivir-selected lineages from earlier passages. We found that this mutation was present as early as the ninth passage in one remdesivir-selected lineage with a SNV frequency of ∼40% (RDV-2, Fig. 1*D*). At the 13th passage, the F548S mutation was detected in lineages RDV-1 and RDV-2 at frequencies > 90%, and by the 23rd passage the mutation was found in five of six remdesivir-selected lineages at frequencies >92% (Fig. 1*D* and *SI Appendix*, Table S1). Multiple sequence alignments of L polymerase amino acid coding sequences indicated that this F548 residue maps to the base of the loop forming motif F within the L polymerase RdRp domain and is conserved across members of the family *Filoviridae* (Fig. 1*E*, cyan box). Other prototypic members of the order *Mononegavirales: Paramyxoviridae*, *Pneumoviridae*, and *Rhabdoviridae* all possess aliphatic amino acids at analogous positions. Aside from the F548S mutation, one other SNV resulted in a T562A mutation, which is also in the F motif; but it was not detected across all six remdesivir-selected lineages. Having emerged after passage 23, this mutation was detected at frequencies between 54 and 73% (Table 2 and Dataset S1).

### Molecular Modeling of RNA-Dependent RNA Polymerase Domains from Ebola Virus and SARS-CoV-2 Indicates a Shared Molecular Region Targeted by Remdesivir.

To visualize the spatial location of the F548 residue and its potential impact on remdesivir activity, we generated an homology model of the EBOV polymerase active site based on structures of human metapneumovirus (hMPV) (14) and respiratory syncytial virus (RSV) L proteins (15). The positions of preincorporated remdesivir triphosphate (RDV-TP) and primer and template RNA strands with respect to the active site were significantly refined from previous models (5) and followed extensive modeling of the replicating state of RSV. We found F548 sits at the N-terminal end of the F-motif in the fingers subdomain, a loop common to polymerase structures that lies over the active site and aids in positioning of both the template base and the substrate triphosphate (Fig. 1*F*, F548 residue is depicted in cyan) (16).

A recent CoV study described two mutations (F480V and V557L) in the RdRp of SARS-CoV-1 that together reduced remdesivir susceptibility by sixfold (17). In view of ongoing clinical trials of remdesivir against COVID-19 (18), we modeled the SARS-CoV-2 replication complex with RDV-TP in its preincorporation state based on the recently published structure of the dsRNA-bound nsp12–nsp7–nsp8 complex (19). While F480 maps to a location in the palm subdomain, V557 maps to the C-terminal end of the F-motif in the fingers subdomain. It is clear that SARS-CoV-2 V557 and EBOV F548 are in a similar part of the active site, but they are not in strictly equivalent positions. The residue most analogous to V557 in EBOV is F563 (Fig. 1 *E*–*G*, V557 residue shown in cyan).

### Evaluation of Ebola Virus L Polymerases Carrying Either the F548S or the T562A Mutation Showed Varying Susceptibilities to Remdesivir Inhibition in a Minigenome Replication System.

**Fig. 2.**
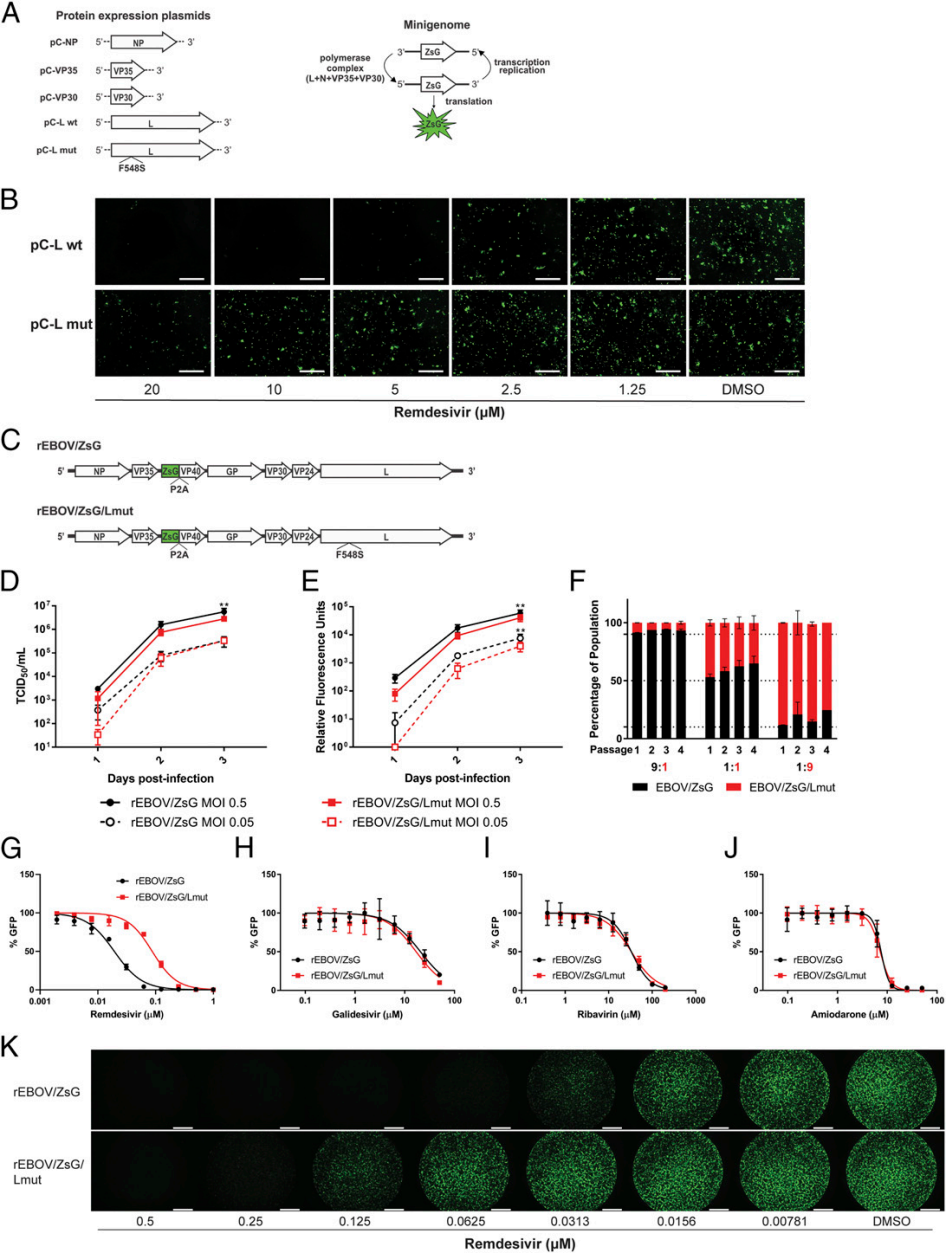
The EBOV L F548S mutation recapitulates reduced susceptibility phenotype against remdesivir. (*A*) Schematic depicting EBOV protein expression plasmids used to make up the polymerase complex within transfected cells to transcribe and replicate an EBOV minigenome encoding the ZsG reporter green fluorescent protein. (*B*) Levels of EBOV minigenome replication as reflected by ZsG expression in transfected Huh7 cells treated with indicated concentrations of remdesivir. DMSO, dimethyl sulfoxide. Fluorescence micrographs taken using 4× magnification; white bars indicate 500 μm. (*C*) Schematic of the full-length rEBOV/ZsG genome alongside an identical genome containing the L polymerase F548S amino acid mutation (rEBOV/ZsG/Lmut). (*D*) Multistep infectious virus titers measured by 50% tissue culture infectious dose (TCID_50_) kinetic growth curves for rEBOV/ZsG and rEBOV/ZsG/Lmut in Huh7 cells using two different multiplicities of infection (MOIs). Error bars indicate SD from the mean of triplicate samples for each time point. (*E*) Multistep infection kinetic curves measuring relative reporter green fluorescence levels emitted by rEBOV/ZsG and rEBOV/ZsG/Lmut infected Huh7 cells using two different MOIs. Error bars indicate SD from the mean of nine replicates for each time point. Two-way analysis of variance with Sidak’s multiple-comparison test was used to measure statistical significance of differences between respective means for *D* and *E*. Double asterisks indicate *P* < 0.01. (*F*) Coinfection competition assay of rEBOV/ZsG and rEBOV/ZsG/Lmut at the indicated ratios. The respective percentages of wild-type (black bars) and F548S (red bars) viral variants present in the population were measured for each of four successive passages. Error bars represent SD from the mean of duplicate samples. Dose–response inhibition curves of rEBOV/ZsG (in black) and rEBOV/ZsG/Lmut (in red) against remdesivir (*G*), galidesivir (*H*), ribavirin (*I*), and amiodarone (*J*). Data are representative of at least five independent experiments of quadruplicate samples per experiment. Error bars indicate SD from the mean of quadruplicate samples. (*K*) Fluorescence micrographs of rEBOV/ZsG- or rEBOV/ZsG/Lmut-infected Huh7 cells treated with serial dilutions of remdesivir or dimethyl sulfoxide (DMSO) control taken 72 hpi at 2× magnification; white bar indicates 1,000 μm. Concentrations of remdesivir are indicated below the micrographs.

Given the uniform prevalence of the F548S mutation across all six remdesivir selected lineages, we first evaluated the EBOV L F548S mutation in the context of a plasmid transfection cell-based EBOV minigenome fluorescence assay in which levels of ZsG green fluorescence expression correlate with levels of minigenome transcription and replication (Fig. 2*A*) (20). Whereas we visibly observed remdesivir-mediated inhibition of minigenome replication driven by wild-type EBOV L (pC-L wt) at a remdesivir concentration of 5 µM, minigenome replication driven by the mutant EBOV L containing the F548S mutation (pC-L mut) was only partially inhibited at the highest concentration (20 µM) of remdesivir used (Fig. 2*B*). At such high concentrations, the inhibition could be attributed to remdesivir cytotoxicity (4).

In addition, since the T562A mutation was detected in four out of six passage 35 remdesivir selected lineages at SNV frequencies >54% and is in spatial proximity to F548S and F563 (*SI Appendix*, Fig. S2*A*), we investigated T562A alone and also in tandem with F548S in the minigenome assay to determine whether it may contribute to the small but measurably comparative reduction in remdesivir susceptibility between lineages possessing T562A (RDV-1, -2, -3, and -5) versus those that only carrying F548S (RDV-4) (Table 1). We observed a twofold reduction in remdesivir susceptibility for T562A alone compared to wild type but could not observe any qualitative difference in minigenome replication between polymerases carrying either F548S or both mutations (*SI Appendix*, Fig. S2*B*) at up to 10 µM of remdesivir. Although T562A occurs naturally in related species such as Bundibugyo virus (BDBV) and Tai Forest virus (TFV) (Fig. 1*E*), we were unable to observe any difference in susceptibility to remdesivir inhibition between infectious wild-type BDBV and EBOV (*SI Appendix*, Fig. S2*C*).

### L F548S Mutant Polymerase Marginally Reduces Selectivity of Remdesivir Incorporation in Cell-Free Primer-Extension Assay.

The inhibitory effects of remdesivir have also been studied in cell-free biochemical assays with the purified RdRp complex (12). While the active form of remdesivir (remdesivir-TP) competes with ATP for incorporation into the growing RNA chain and causes delayed chain termination, steady-state kinetics for single-nucleotide incorporations revealed that ATP is the preferred substrate. The efficiency of incorporation *V*_max_/*K*_m_ (ATP) over *V*_max_/*K*_m_ (remdesivir-TP) previously yielded a selectivity value of ∼4. Here, we compared selectivity measurements of the wild-type RdRp complex with the F548S mutant in a sequence context that also allowed us to compare inhibition patterns (*SI Appendix*, Fig. S3). For the mutant, we measured a selectivity of 7.3, and for the wild type we measured a slightly lower value of 5.1 under the same conditions. Selectivity (F548S)/selectivity (wild type) is 1.4 and provides a measure for the resistant phenotype (*SI Appendix*, Fig. S3*A*). The subtle difference of 1.4 is mainly driven by differences in the *K*_m_ value for remdesivir-TP incorporation. Using the same primer/template substrate, we also analyzed possible changes in patterns of inhibition but did not observe any differences in RNA synthesis nor inhibition patterns between wild type and the mutant (*SI Appendix*, Fig. S3*B*).

### Incorporation of L F548S Mutation into Full-Length rEBOV/ZsG Does Not Significantly Alter Viral Fitness.

To investigate the effect of the L F548S mutation in the context of an infectious virus, we used reverse genetics to generate a recombinant full-length cDNA clone of a mutant rEBOV/ZsG virus carrying the F548S substitution (rEBOV/ZsG/Lmut) (Fig. 2*C*). Upon successful rescue of infectious rEBOV/ZsG/Lmut virus in cell culture, we conducted multistep growth kinetic curves for rEBOV/ZsG and rEBOV/ZsG/Lmut, using infectious virus yield and relative fluorescence signal as quantitative measures for growth (Fig. 2 *D* and *E*). Both measures found similar growth kinetics between the two, with wild-type rEBOV/ZsG only attaining a modestly higher peak titer at the latest time point of infection (∼72 h postinfection [hpi]). The rEBOV/ZsG/Lmut had comparatively lower levels of reporter fluorescence levels throughout the infection time course than wild-type rEBOV/ZsG (Fig. 2*E*), suggesting that the L F548S mutation may confer a subtle reduction in viral fitness. To examine this possibility, we serially copassaged three different mixtures of rEBOV/ZsG and rEBOV/ZsG/Lmut using wild type:mutant ratios of 9:1, 1:1, and 1:9 based on their respective viral stock titers; experiments were conducted in duplicate. At the end of each passage, we quantified viral RNA present in supernatants and measured the percentage of rEBOV/ZsG versus rEBOV/ZsG/Lmut present in the total virus population by NGS. For every mixture ratio tested and at every ensuing passage, we observed relatively stable allele frequencies with marginal increases observed in the percentage of wild-type rEBOV/ZsG sequence; certain instances of potential random genetic drift could be seen in passage 3 of the 1:9 ratio mixture (Fig. 2*F*). Furthermore, we conducted five serial passages of passaged rEBOV/ZsG/Lmut in the absence of remdesivir selection, and observed that prevalence of the F548S mutation for each passage remained >99.7% (*SI Appendix*, Table S2).

### Incorporation of L F548S Mutation into Full-Length rEBOV/ZsG Confers Fourfold Reduced Susceptibility Specific to Remdesivir.

To determine whether the L F548S mutation conferred a specific advantage against remdesivir, we tested remdesivir along with two other known nucleoside analog inhibitors of EBOV replication (galidesivir, ribavirin) (21, 22), and amiodarone, a repurposed antiarrhythmic drug known to block EBOV entry (23) against rEBOV/ZsG and rEBOV/ZsG/Lmut. We observed that while the dose–response curves for rEBOV/ZsG/Lmut recapitulated the fourfold reduced susceptibility phenotype observed across all passage 35 remdesivir-treated viruses (Fig. 2 *G* and *K* and Table 3), the dose–response curves for galidesivir, ribavirin, and amiodarone showed near-equivalent inhibition of rEBOV/ZsG and rEBOV/ZsG/Lmut (Fig. 2 *H*–*J* and Table 3).

**Table 3.** Comparative susceptibilities of rEBOV/ZsG and rEBOV/ZsG/Lmut to antiviral agents

	rEBOV/ZsG	rEBOV/ZsG/Lmut	
Compound ID	compound EC_50_, µM	compound EC_50_, µM	Fold change over EBOV/ZsG
Remdesivir	0.021 ± 0.001	0.086 ± 0.007	4.10
Galidesivir	20.3 ± 2.8	16.4 ± 6.2	0.81
Ribavirin	29.7 ± 4.2	31.4 ± 4.0	1.06
Amiodarone	7.2 ± 0.48	6.0 ± 0.82	0.83

Mean EC_50_ values were calculated from reporter assays conducted 72 hpi. Means and SDs are derived from at least five independent experiments consisting of four biological replicates per experiment.

## Discussion

With the increasing emergence and reemergence of zoonotic viral pathogens with pandemic potential over the past several decades and into the present COVID-19 pandemic (24), development of broad-spectrum antiviral therapeutics is now needed more than ever. We have recently demonstrated the broad-spectrum in vitro antiviral activity of remdesivir across three virus families, as well as its in vivo efficacy against both EBOV and NiV in nonhuman primates (3, 4, 9). The active nucleotide triphosphate (NTP) form of remdesivir has also been shown to biochemically inhibit the RdRp activities of EBOV, NiV, and respiratory syncytial virus (3, 12, 25), and more recently also those of MERS-CoV, SARS-CoV-1, and SARS-CoV-2 (26, 27). In this study, we showed that serial passaging of rEBOV/ZsG under subclinical remdesivir concentrations resulted in the emergence of a nonsynonymous substitution F548S near the base of conserved motif F, which forms a channel allowing for NTP entry (14). While this mutation correlated with a fourfold to fivefold reduction in susceptibility of EBOV/ZsG to remdesivir, it was still susceptible to remdesivir inhibition at higher concentrations. Using reverse genetics, we confirmed that the F548S mutation reduced susceptibility against remdesivir, with an additional caveat that a T562A mutation may modestly contribute to further reduction in remdesivir susceptibility only when accompanied by a preexisting F548S mutation.

The cell-free biochemical kinetic measurements we conducted only showed a subtle difference between wild-type and mutant polymerases. In general, biochemical assays used to determine a possible effect of resistance conferring mutations may not adequately capture conditions in a cellular environment. Our selectivity measurements are based on single-nucleotide incorporations, while viral replication and transcription involve several thousands of these events; hence, the effect of a mutant could be stronger under biologically relevant conditions. It is also conceivable that the resistance-conferring effect is not or not only mediated at the level of nucleotide incorporation. Future studies should investigate other potential mechanisms of action for remdesivir such as template-mediated inhibition, as has been shown recently for SARS-CoV-2 RdRp (39). Given the complete conservation of the F548 residue across filoviruses, it is reasonable to infer that a nonconservative change from a neutral aromatic amino acid to a polar amino acid near the enzymatic catalytic domain could cause a large-scale disruption of protein structure and function. However, given that the serine substitution did not significantly affect viral fitness, it is more likely that the F548S mutation alters peripheral hydrophobic interactions in the fingers domain that govern the positioning of the RNA template and NTP substrate in the active site.

The potential for novel filoviruses to emerge and cross over into humans is of obvious public health concern. While the F548 residue is completely conserved across known filoviruses, this may not be true for newly emergent sequences or for variants that arise amid ongoing outbreaks. As such variants may reduce the effectiveness of remdesivir, or of other mechanistically similar nucleotide analogs that may be in development, we suggest that substitutions at the polymerase F548 position, particularly F548S, be added alongside the watch list of antibody escape mutations in the EBOV glycoprotein (28, 29) and be subject to particular attention during surveillance.

We found that the F548S substitution did not affect susceptibility to inhibition by several other EBOV inhibitors. Amiodarone blocks EBOV entry and a change in the polymerase would not be expected to alter susceptibility, but galidesivir and ribavirin are both nucleoside analogs. Their equivalent potency against wild type and the F548S variant suggest that either their activities may be mechanistically distinct from remdesivir, or at least that they interact with the EBOV polymerase in a different manner. Thus, combining remdesivir with either galidesivir or ribavirin (or both) may improve antiviral efficacy by way of reducing the likelihood of emerging drug resistance.

Since remdesivir has recently received EUA for the treatment of COVID-19, we were interested to see how our findings compared with those for coronaviruses. Both mouse hepatitis virus (MHV) and SARS-CoV-1 had fivefold to sixfold reduced susceptibilities to remdesivir when containing two nonsynonymous mutations in their respective RdRps (17). Interestingly, one of these mutations (V553L in MHV, corresponding to V557L in SARS-CoV-1 and SARS-CoV-2) also mapped to the F motif and by itself was able to reduce remdesivir susceptibility of MHV by fivefold (17). Thus, remdesivir is sensitive to mutations in a similar region of the EBOV and SARS-CoV-1 polymerases, and this likely also applies to SARS-CoV-2 as well as other susceptible virus families in the order *Mononegavirales* (4, 12, 25, 30). While the EBOV residue F548 is not strictly equivalent to SARS-CoV-2 residue V557, we might hypothesize that mutations of these two residues elicit a similar effect on RDV-TP incorporation. Specifically, the V557 equivalent residue in EBOV is F563. In SARS-CoV-2, V557 is seen to present a hydrophobic surface to the template base, and F563 is predicted to serve the same role in EBOV. Our model further shows that F548 is in direct contact with G564, such that the F548S mutation is bound to alter the position of G564 and its neighboring residues. As argued elsewhere (30), the leading hypothesis for the impact of the V557L mutation is to alter the position of the template base. We infer that remdesivir, with its 1′CN substitution, is more sensitive to this template repositioning than ATP. We suggest here that a similar situation is created with the F548S mutation in EBOV.

Remdesivir was recently evaluated for efficacy against acute EVD as part of the Pamoja Tulinde Maisha clinical trial in the DRC, and while not as efficacious as two antibody-based treatments, survival in remdesivir-treated patients was higher compared to the overall mortality rate of the epidemic (1). Although the clinical trial regimens of i.v. remdesivir treatment normally span 5 to 14 d, it is difficult to estimate the likelihood of reduced susceptibility mutations arising in remdesivir-treated patients. Moreover, sequencing EBOV from remdesivir-treated rhesus macaques challenged with EBOV did not reveal any notable mutations (3). Retrospective surveillance of EBOV sequences from remdesivir-treated EVD patients for amino acid changes in the polymerase would be useful to determine the potential for emerging resistance to remdesivir treatment. Given the ongoing clinical evaluation of remdesivir to treat COVID-19 (18), SARS-CoV-2 sequences from remdesivir-treated participants should similarly be monitored.

## Materials and Methods

### Biosafety.

This project was conducted with review and approval from CDC’s Institutional Biosecurity Board and the Federal Select Agent Program. All work with live infectious recombinant EBOV was conducted in the biosafety level 4 (BSL-4) high containment laboratory at the US CDC. Experiments involving cDNA encoding viral sequences were performed in accordance with approved Institutional Biosafety Committee protocols. Minigenome experiments were conducted under BSL-2 containment.

### Compounds.

Remdesivir was synthesized at Gilead Sciences, Inc., and prepared as 10 or 20 mM stock solutions in dimethyl sulfoxide (DMSO) as previously described (3, 4).

### Cell Culture.

Human hepatoma (Huh7) (Apath, LLC) cells were cultivated in Dulbecco’s modified Eagle medium supplemented with 7.5% fetal bovine serum (FBS), nonessential amino acids (NEAA), and penicillin/streptomycin. For reporter virus assays, cells were seeded using in Fluorobrite medium (Thermo Fisher) supplemented with 7.5% FBS, NEAA, 1% glutamax, and penicillin/streptomycin. Cells were incubated at 37 °C and 5% CO_2_.

### Viruses and Plasmid Cloning.

Wild-type rEBOV/ZsG (recombinant Makona variant) and mutant rEBOV/ZsG carrying the single F548S substitution (EBOV/ZsG/Lmut) were rescued from cDNA, propagated using Huh7 cells, and quantitated by determining TCID_50_ as previously described (13). Briefly, Huh7 cells grown in 12-well plates were transfected with 1 μg of pEBOV, 0.5 μg of pC-L, 0.5 μg of pC-NP, 0.05 μg of pC-VP35, 0.05 μg of pC-VP30, and 1 μg of codon-optimized pC-T7. EBOV/ZsG/Lmut was generated from EBOV/ZsG cDNA that was modified by PCR-based site-directed mutagenesis. Similarly, site-directed mutagenesis of a eukaryotic expression plasmid encoding the EBOV L polymerase (pc-L) was used to create a mutant L plasmids encoding either F548S substitution (pC-Lmut), the T562A mutant, or a double mutant encoding both changes.

### Selection of rEBOV/ZsG with Reduced Remdesivir Susceptibility.

A total of 5 × 10^5^ Huh7 cells per well was seeded in six-well plates and were infected with wild-type recombinant rEBOV/ZsG virus using a multiplicity of infection (MOI) of 1 for 1 h. After a 2-mL wash with PBS, cells were replenished with 2 mL of Fluorobrite media supplemented as mentioned above with 0.1 to 0.2 µM remdesivir (*n* = 6) or without remdesivir (mock-treated) (*n* = 2), and incubated for 72 h prior to collecting virus supernatants for infectious virus titer determination and for RNA sequencing. The volumes of supernatant used for ensuing serial passaging ranged between 5 and 1,000 µL. The following passages were conducted in the absence of remdesivir (*n* = 2) or the presence of subclinical concentrations (0.01 to 0.16 µM) of remdesivir (*n* = 6); cells were incubated between 72 and 144 h postinfection before supernatant harvest depending on the progression of infection as indicated by prevalence of visible ZsG expressing cells by fluorescence microscopy.

### Reporter Virus Assay and Fluorescence Microscopy.

Levels of recombinant EBOV/ZsG replication in all dose–response inhibition assays were determined by measuring green fluorescence expression using an HD1 Synergy plate reader (Biotek) as previously described (4). EC_50_ values were calculated using four-parameter variable slope nonlinear regression fitting of mean values of assays performed in duplicate or quadruplicate. Huh7 cells were seeded at 10^4^ cells per well in black opaque or black-sided, clear-bottomed 96-well plates, and remdesivir was added. Assay plates were then transferred to the BSL-4 suite and infected with 0.25 TCID_50_ per cell of EBOV/ZsG viruses; fluorescence was read between 72 and 144 hpi. All fluorescence micrographs were taken of ZsG-positive cells using an EVOS imager (Thermo Fisher) at either 2× or 4× magnification using a gain between 30 and 40%.

### EBOV Minigenome Assay.

A ZsG green fluorescence reporter-based EBOV minigenome assay (20) was used as follows: Huh7 cells (100,000 per well) were seeded in 24-well plates and transfected with appropriate amounts of EBOV support plasmids (0.25 ng/well NP, 0.025 ng/well VP35, 0.025 ng/well VP30, and 0.5 ng/well L or Lmut), and EBOV ZsG minigenome (0.75 ng) prepared in RNase-free Tris-EDTA buffer mixed with 0.9 μL/well LT-1 transfection reagent (MirusBio) and 10 μL of Opti-MEM per well. Complexes were incubated for 30 min at room temperature before adding to cells. Compounds were added directly to the cells 4 h post transfection. For negative controls, the L plasmid was substituted with an equivalent inactive L plasmid. At 48 to 72 h post transfection, fluorescence micrographs were taken of ZsG-positive cells.

### Quantitative Focus-Forming Unit Assay.

To measure compound inhibition of filovirus infection and spread, 1 × 10^4^ Huh7 cells pretreated with remdesivir for 1 h were infected with 0.25 TCID_50_ of EBOV (Makona variant) or BDBV (13, 31). At 3 d pi, cells were fixed in 10% formalin supplemented with 0.2% Triton-X detergent, and stained with primary rabbit anti-EBOV polyclonal serum (1:1,000), and after several washes the corresponding anti-rabbit Dylight 488 conjugated secondary antibody (Bethyl Laboratories) was added (1:1,000) for 1 h. After three washes, filovirus infection-induced focus-forming units (FFUs) were measured in each well of the 96-well opaque black plate with clear bottoms (Costar, 3603) using a Cytation5 cell imaging multimode reader paired with Gen5 software (Biotek). A 2.74× objective lens was used to take 12 overlapping images encompassing each entire well, which were assembled and then analyzed for FFUs ranging in size from 20 to 400 µm, and which had a relative fluorescence signal that was above 5,000. The camera gain was set at 15.6, with an integration time of 306 ms, using an LED intensity setting of 10. The average number of background cell counts were subtracted from each well to give normalized cells counts, and any negative values were adjusted to “0.” For analysis of each experimental replicate, the highest number of positive counts was regarded as 100%, while 0 count was used for 0% positivity. Following this normalization, data were fitted to a four-parameter variable slope nonlinear regression fitting of mean values derived from quadruplicate samples.

### Protein Expression, Purification, and RNA Synthesis Assays.

The pFastBac-1 (Invitrogen) plasmid with the codon-optimized synthetic DNA sequences coding for EBOV polymerase complexes (GenScript) was used for viral protein expression in insect cells (Sf9; Invitrogen) as previously described (12). The construct for the production of the mutant F548S EBOV RdRp (L:VP35) was generated with a mutagenic primer. The codon for the amino acid substitution was introduced with Phusion High-Fidelity DNA polymerase (Thermo Fisher Scientific) according to the manufacturer’s recommendations. The sequence was confirmed at Molecular Biology Facility at University of Alberta, Canada. We employed the MultiBac (Geneva Biotech) system as previously described (32). Viral proteins and protein complexes were purified using the His- or Strep tag-affinity chromatography according to the manufacturer’s specifications (IBA and Thermo Fisher Scientific, respectively). The overall yield of functional partially purified EBOV RdRp (100 to 150 μL per 3 L of insect cell culture) precluded additional purification steps. Instead, we applied stringent purification conditions, which included extensive column washes (60 column volumes) in the presence of high salt and the use of 100-kDa molecular weight cutoff membranes to concentrate the protein preparation. The identity of the purified viral proteins and protein complexes was confirmed by mass spectrometry (MS) analysis with data processing and analysis being performed using Proteome Discoverer 1.4 and Sequest (Thermo Fisher Scientific), respectively (32).

### Multiple Sequence Alignment and Modeling of EBOV L Polymerase; Modeling of SARS-CoV-2 nsp12 RdRp.

A multiple sequence alignment of the L polymerase coding sequences from viruses within the order *Mononegavirales* was generated using the Clustal W alignment tool in the MEGA 7 software (33). Based on the determined structures of the L polymerases of hMPV (14), RSV (15), SARS-CoV-1 (34), and SARS-CoV-2 (19), further alignment of conserved motif F was conducted to include the abovementioned coronavirus RdRps. The alignment was then visualized using the ESPript 3.0 software (35). The accession numbers for the L polymerase coding sequences used in this alignment are listed in the following: human metapneumovirus (hMPV) (GenBank accession number Q6WB93), Ebola virus (EBOV) (AKI82631), Tai Forest virus (TFV) (YP_003815431), Bombali virus (BOMV) (YP_009513282), Bundibugyo virus (BDBV) (AYI50387), Sudan virus (SUDV) (AWK96585), Reston virus (RESTV) (NP_690587), Lloviu cuevavirus (LCV) (YP_004928143), Mengla virus (MLAV) (AZL87829), Marburg virus (MARV) (Angola) (AKI84286), Marburg virus (RAVN) (YP_009055228), human parainfluenza virus 3 (hPIV-3) (NP_067153), measles virus (AAR32667), mumps virus (AEI98832), Nipah virus (NiV) (AAY43917), respiratory syncytial virus (RSV) (YP_009518860), vesicular stomatitis virus (VSV) (Q98776), Middle East respiratory syndrome coronavirus (MERS-CoV) (YP_009047223), severe acute respiratory syndrome coronavirus (SARS-CoV-1) (6NUR_A), and SARS-CoV-2 (YP_009725307). The putative structure of the EBOV L polymerase in its replicating state was modeled based on the defined structures of the human metapneumovirus (PDB ID code 6U5O) (14) and respiratory syncytial virus (PDB ID code 6PZK) (15) polymerases. The model of preincorporated RDV-TP in SARS-CoV-2 nsp12 was based on the defined structure of the SARS-CoV-2 replication complex nsp12–nsp7–nsp8 with dsRNA (PDB ID code 6YYT) (19). The model was generated with the Schrodinger 2020-2 suite of software (36) and represents an update of a previous model based on earlier CoV structures (30).

### Virus Inactivation, RNA Extraction, Ribosomal RNA Depletion, NGS, and Analysis.

One milliliter of supernatants from EBOV/ZsG-infected cells from selected serial passages was inactivated with 4 mL of TRIPURE (Roche) reagent and removed from the BSL-4 laboratory. Total RNA from these samples was extracted using the Direct-zol RNA purification kit (Zymo Research). For passage 35 samples, ribosomal RNA was removed and cDNA libraries were prepared using the NEBNext rRNA depletion kit (New England BioLabs) in tandem with the TruSeq Stranded mRNA kit (Illumina), following a modified version of the manufacturer’s protocol that omits steps related to mRNA purification. For all other passages and experiments, we used the KAPA RNA HyperPrep Kit with RiboErase (HMR) (Roche) for improved sequence coverage and quality. NGS was performed using paired-end 2 × 150-bp chemistry on an Illumina MiniSeq instrument. The NGS sequences were analyzed using Gilead’s internal analysis pipeline. Sequence reads were trimmed based on low quality, phred <15 in 4-bp sliding window, and filtered on sequence length (<50 base pairs) with Trimmomatic (37). Paired-end reads that overlap were merged using PEAR software (38). Merged and unmerged reads were aligned to the rEBOV/ZsG reference sequence (GenBank accession number: KR781609.1) using the SMALT aligner [SMALT is copyright © 2010–2015 Genome Research Ltd.]. All bases within 15 bases of the start or end of read were excluded from variant calling and any variant with phred score <30 was excluded from analysis. If a read contained indel(s), the read was evaluated for number of insertions and deletions. If sum of insertions minus sum of deletions was a multiple of 3, amino acid alignment was performed to realign indels. If not a multiple of 3, we either extracted the longest fragment between two indels, the 5′ end to indel, or from the indel to the 3′ end. Frameshift insertions and deletions were also excluded.

### Competitive Fitness of EBOV/ZsG and EBOV/ZsG/Lmut.

A total of 5 × 10^5^ Huh7 cells was coinfected with recombinant EBOV/ZsG and EBOV/ZsG/Lmut viruses at initial input ratios of 9:1, 1:1, or 1:9 at an MOI of 0.25 TCID_50_/cell for 1 h, after which the inoculum was removed and cells were replenished with fresh medium. At 72 hpi, virus-containing cell supernatants were collected and inactivated with TRIPURE as described above. cDNA library construction, NGS, and analyses were conducted as described above. Twenty microliters of collected virus supernatant were used for infection in the following passage. Results represent the frequencies of wild-type F548 versus the F548S mutation present in the mixed population of viral RNA.

### Statistical Analysis.

Statistical tests were performed using GraphPad Prism 8 software as described in Fig. 2 *D* and *E*. Two-way analysis of variance with Sidak’s multiple comparison test was used to measure statistical significance of differences between respective means for (*D*) (*n* = 3) and (*E*) (*n* = 9). For *D*, MOI = 0.5: *T* values and degrees of freedom, respectively, are as follows: day/row 1, 0.002468, 12; day/row 2, 1.099, 12; day/row 3, 3.721, 12. MOI = 0.05: day/row 1, 0.006113, 12; day/row 2, 0.3209, 12; day/row 3, 0.03192, 12. For *E*, MOI = 0.5: *T* values and degrees of freedom, respectively, are as follows: day/row 1, 0.05177, 48; day/row 2, 2.007, 48; day/row 3, 4.484, 48. MOI = 0.05: day/row 1, 0.01039, 48; day/row 2, 1.928, 48; day/row 3, 5.937, 48.

## Supplementary Material

Supplementary File

Supplementary File

Supplementary File

## Data Availability

The raw data used for the manuscript figures has been submitted as Dataset S1. A master list of all SNVs detected at frequencies >15% across all lineages and all serial passages sequenced has been submitted as Dataset S2. NGS raw sequencing files are available upon request and have been deposited to National Center for Biotechnology Information Sequence Read Archive under BioProject number PRJNA664887.
