# Supplementary material for: Remdesivir targets a structurally analogous region of the Ebola virus and SARS-CoV-2 polymerases
Source: Proc Natl Acad Sci U S A. 2020 Oct 7;117(43):26946–54. doi: 10.1073/pnas.2012294117 (PMC7604432; doi:10.1073/pnas.2012294117)
Supplement: Supplementary File [file pnas.2012294117.sapp.pdf]

PNAS
www.pnas.org

Supplementary Information for

Remdesivir targets a structurally analogous region of the Ebola virus and SARS-CoV-2 polymerases

Michael K. Lo[1#], César G. Albariño[1], Jason K. Perry[2], Silvia Chang[2], Egor P. Tchesnokov[3,4], Lisa Guerrero[1], Ayan Chakrabarti[1], Punya Shrivastava-Ranjan[1], Payel Chatterjee[1], Laura K. McMullan[1], Ross Martin[2], Robert Jordan[2*], Matthias Götte[3,4], Joel M. Montgomery[1], Stuart T. Nichol[1], Mike Flint[1], Danielle Porter[2], Christina F. Spiropoulou[1#]
[1] Viral Special Pathogens Branch, US Centers for Disease Control and Prevention, 1600 Clifton Road NE, Mailstop H18-SB, Atlanta, GA, 30329, USA
[2] Gilead Sciences Inc., Foster City, CA, 94404, USA
[3] Department of Medical Microbiology and Immunology, University of Alberta, Edmonton, AB T6G 2E1, Canada.
[4] Li Ka Shing Institute of Virology at University of Alberta, Edmonton, AB T6G 2E1, Canada

[*] Current address: Research and Development, Meissa Vaccines, Inc., South San Francisco, CA, 94080, USA

Christina F. Spiropoulou, Michael K. Lo
Email: ccs8@cdc.gov, mko2@cdc.gov.

This PDF file includes:

Figures S1 to S3
Tables S1 to S2

SI References

Other supplementary materials for this manuscript include the following:

Datasets S1 to S2

www.pnas.org/cgi/doi/10.1073/pnas.2012294117

S1.

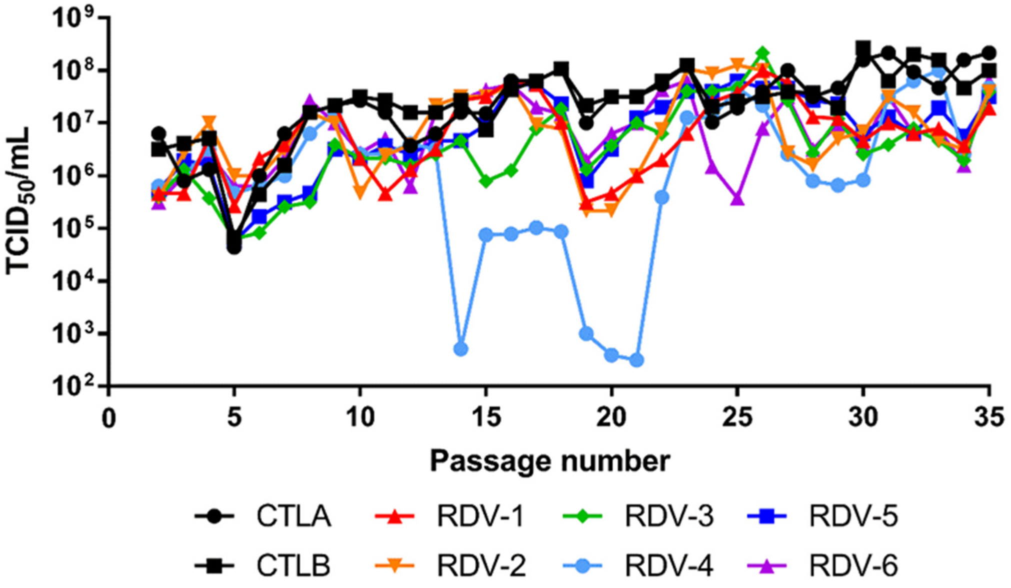

Fig. S1. Infectious titer of rEBOV/ZsG lineages according to passage number. Infectious virus titer of mock-selected and remdesivir-selected rEBOV/ZsG lineages were categorized in order of serial passage. Infectious virus titer was measured by $TCID_{50}$ assay. Mock-selected lineages are indicated by black shapes and lines; remdesivir-selected lineages are indicated by colored shapes and lines.

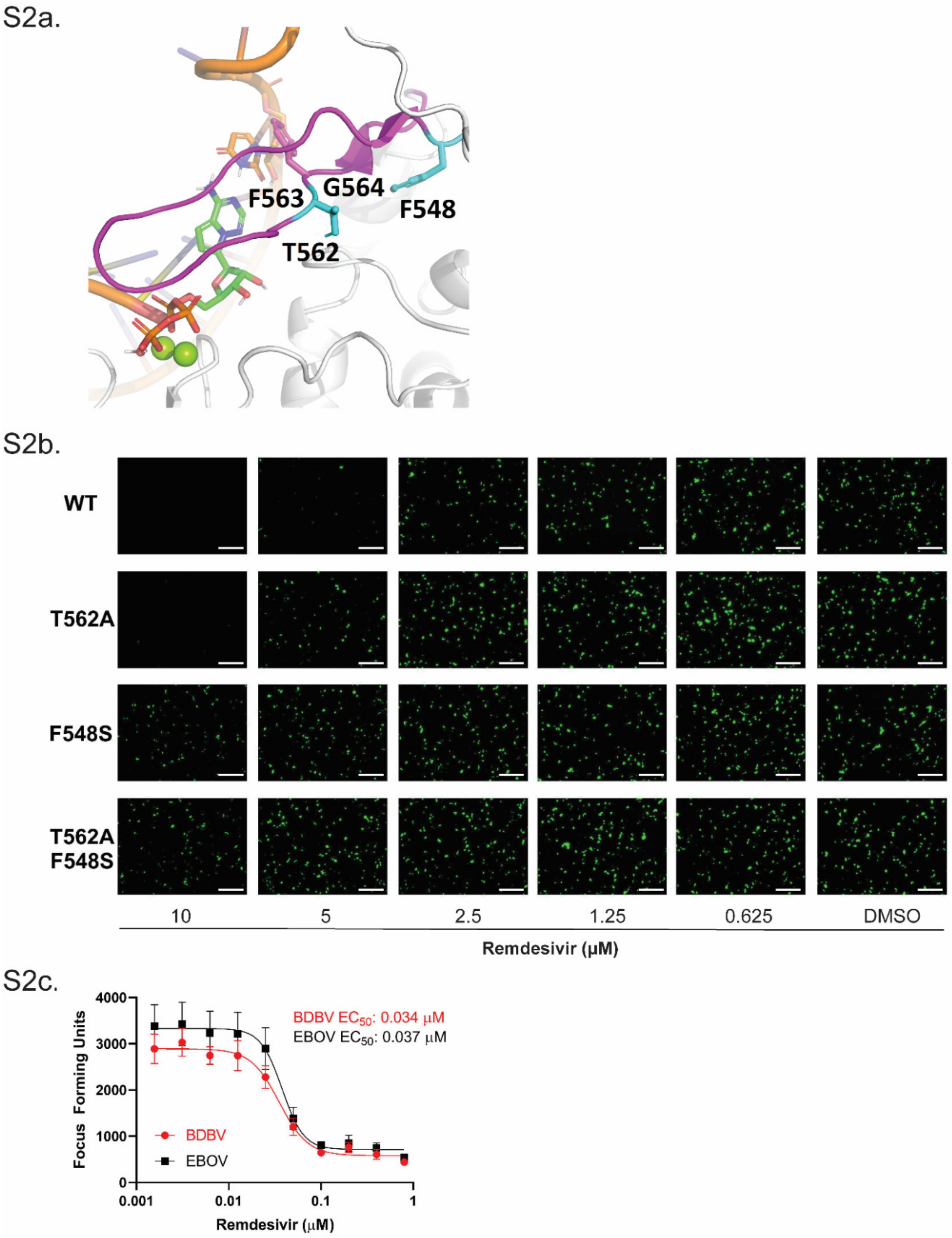

Fig. S2. Visualization and phenotypic evaluation of EBOV L T562A mutation in minigenome and infectious virus assays. (a) Structural modeling of conserved motif F of EBOV L polymerase in

complex with remdesivir-TP. Motif F is colored in violet. Side groups of amino acid positions F548, T562, F563, and G564 and are visible. (b) Levels of EBOV minigenome replication as reflected by ZsG expression in transfected Huh7 cells treated with indicated concentrations of remdesivir. EBOV L coding sequences bearing specific mutations are indicated on the left. DMSO- dimethylsulfoxide. Fluorescence micrographs taken using 4× magnification; white bars indicate 500 microns. (c) Infectious focus forming unit assay. Huh7 cells pretreated with serial dilutions of remdesivir were infected with either wild-type EBOV (black) or BDBV (red) for 72 h. Virus antigen positive foci were stained and quantitated, with respective EC_{50} values shown for each virus shown. Graph is representative of two independent experiments conducted in quadruplicate for each virus at each remdesivir concentration.

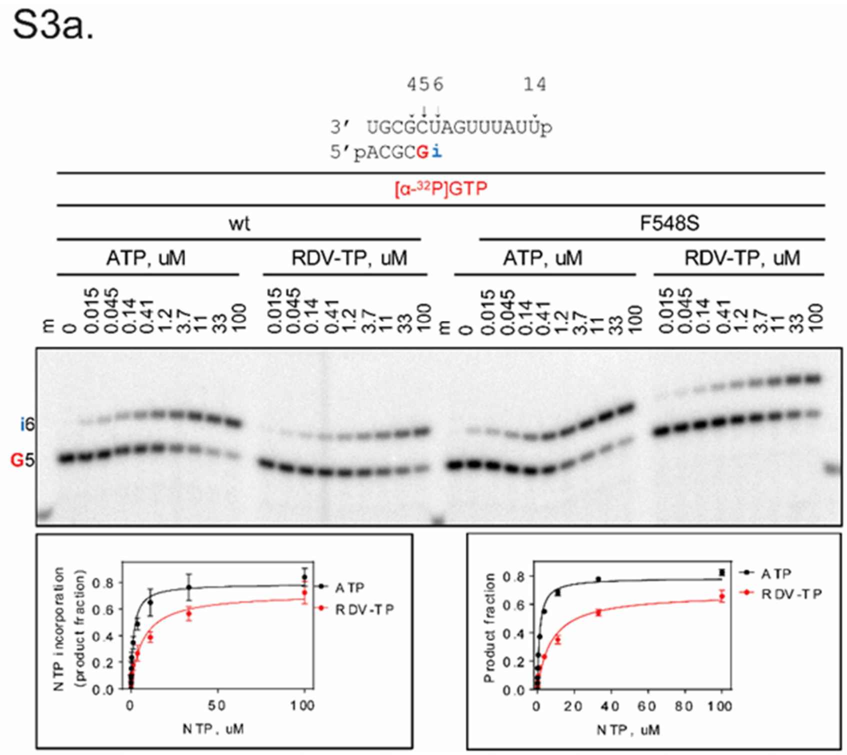

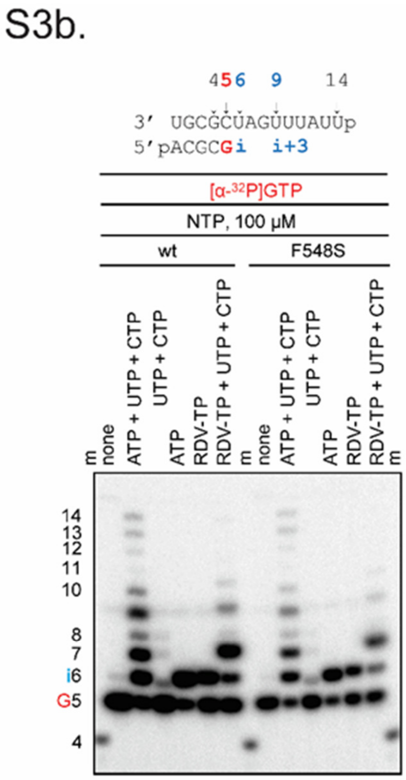

EBOV RdRp	wt			F548S	
	ATP	RDV-TP		ATP	RDV-TP
Vmax (product fraction)	0.80	0.70		0.78	0.68
	0.021	0.029		0.017	0.025
Km (uM)	1.3	5.8		1.2	7.6
	0.17	0.9		0.12	1
Vmax/Km	0.62	0.12		0.65	0.09
Selectivity		5.1			7.3
Resistance					1.4

Figure S3. Incorporation and inhibition of remdesivir-TP in cell-free, biochemical assays with EBOV wild-type and F548S mutant polymerases. (a) Selective incorporation of ATP over remdesivir-TP was evaluated using a short model primer/template as previously described (1). Efficiency of single nucleotide incorporations were monitored in the presence of [a-^{32}P]GTP (position 5) and increasing concentrations of ATP or remdesivir-TP (position 6). Steady-state kinetic parameters V_{max} and K_m reflect the maximal velocity of nucleotide incorporation and the concentration of the nucleotide substrate at which the velocity of nucleotide incorporation is half of Vmax, respectively. Efficiency of nucleotide incorporation is defined as V_{max}/K_m. Selectivity is calculated as the ratio of the V_{max}/K_m values for ATP and remdesivir-TP. Resistance is defined as selectivity of the mutant enzyme over selectivity of the wild-type enzyme. Experiment have been performed three times and the standard error (±) associated with the fit is provided underneath the V_{max} and K_m values. (b) Delayed chain-termination was analyzed with the same primer/template. The presence of all four NTPs yields a 14-mer full-length and several shorter

RNA products with both enzymes. The omission of ATP or remdesivir-TP prevents RNA synthesis. A faint band at position 6 suggests mismatch incorporation. Incorporation of ATP and remdesivir-TP, respectively, yields a strong signal at this position. Reactions with remdesivir-TP and the other three NTPs shows delayed chain-termination at position i+3 and i+4, while the full-length product is not seen. The data do not reveal any significant difference between wild-type and mutant enzyme.

Table S1. SNVs resulting in CDS changes detected in remdesivir-selected passage 9, 13, and 23 rEBOV/ZsG lineages that were also detected at least twice across passage 35 remdesivir-selected rEBOV/ZsG lineages.

		**Passage 9**	**Passage 13**	**Passage 23**	**Passage 23**
	**Gene & SNV ID**	L T13984C	L T13984C	VP35 G4056A	L T13984C
**Virus ID**	**CDS change**	F548S	F548S	S310N	F548S
RDV-1	%SNV freq.		90.76		99.49
	read depth		357		2920
RDV-2	%SNV freq.	41.9	96.39	34.6	99.91
	read depth	2456	581	10044	2296
RDV-3	%SNV freq.				99.84
	read depth				2449
RDV-4	%SNV freq.				
	read depth				
RDV-5	%SNV freq.				99.7
	read depth				2297
RDV-6	%SNV freq.			15.72	92.89
	read depth			12929	2012

Reference sequence for rEBOV/ZsG: GenBank accession number: KR781609.1. Freq.- frequency; Cutoff frequency for SNV detection: 15%.

Table S2. Single nucleotide frequency (SNV) of amino acid coding sequence position F548S in rEBOV/ZsG/Lmut across 5 serial passages without remdesivir selection.

	**Gene & SNV ID**	L T13984C
**Virus ID**	**CDS change**	F548S
rEBOV/ZsG/Lmut	%SNV freq.	99.9
	read depth	2093
Passage 1	%SNV freq.	99.94
	read depth	3243
Passage 2	%SNV freq.	99.94
	read depth	1778
Passage 3	%SNV freq.	99.82
	read depth	4528
Passage 4	%SNV freq.	99.77
	read depth	2204
Passage 5	%SNV freq.	99.74
	read depth	1923

Reference sequence for rEBOV/ZsG: GenBank accession number: KR781609.1. Freq.-frequency; Cutoff frequency for SNV detection: 15%.

SI References

1. Tchesnokov EP, Feng JY, Porter DP, & Gotte M (2019) Mechanism of Inhibition of Ebola Virus RNA-Dependent RNA Polymerase by Remdesivir. *Viruses* 11(4).

Dataset S1 (Data File S1.xlsx). Raw data for manuscript figures 1-2 as indicated by annotated tabs.

Dataset S2 (Data File S2.xlsx). Master list of SNVs observed at frequencies greater than 15% for EBOV L, NP, VP35, and VP30 across all sequenced serial passages for 2 mock and 6 remdesivir selected lineages.
